# A Very Low Power MAC (VLPM) Protocol for Wireless Body Area Networks

**DOI:** 10.3390/s110403717

**Published:** 2011-03-25

**Authors:** Niamat Ullah, Pervez Khan, Kyung Sup Kwak

**Affiliations:** Graduate School of Information & Communication Engineering, Inha University, 253 Yonghyun-dong, Nam-gu, Incheon 402-751, Korea; E-Mails: pervaizkanju@hotmail.com (P.K.); kskwak@inha.ac.kr (K.S.K.)

**Keywords:** MAC protocol, patient monitoring, WBAN, wakeup radio

## Abstract

Wireless Body Area Networks (WBANs) consist of a limited number of battery operated nodes that are used to monitor the vital signs of a patient over long periods of time without restricting the patient’s movements. They are an easy and fast way to diagnose the patient’s status and to consult the doctor. Device as well as network lifetime are among the most important factors in a WBAN. Prolonging the lifetime of the WBAN strongly depends on controlling the energy consumption of sensor nodes. To achieve energy efficiency, low duty cycle MAC protocols are used, but for medical applications, especially in the case of pacemakers where data have time-limited relevance, these protocols increase latency which is highly undesirable and leads to system instability. In this paper, we propose a low power MAC protocol (VLPM) based on existing wakeup radio approaches which reduce energy consumption as well as improving the response time of a node. We categorize the traffic into uplink and downlink traffic. The nodes are equipped with both a low power wake-up transmitter and receiver. The low power wake-up receiver monitors the activity on channel all the time with a very low power and keeps the MCU (Micro Controller Unit) along with main radio in sleep mode. When a node [BN or BNC (BAN Coordinator)] wants to communicate with another node, it uses the low-power radio to send a wakeup packet, which will prompt the receiver to power up its primary radio to listen for the message that follows shortly. The wake-up packet contains the desired node’s ID along with some other information to let the targeted node to wake-up and take part in communication and let all other nodes to go to sleep mode quickly. The VLPM protocol is proposed for applications having low traffic conditions. For high traffic rates, optimization is needed. Analytical results show that the proposed protocol outperforms both synchronized and unsynchronized MAC protocols like T-MAC, SCP-MAC, B-MAC and X-MAC in terms of energy consumption and response time.

## 1. Introduction

Wireless Sensor Networks (WSNs) have and will continue to play a vital role in our daily lives. A WSN consists of sensor nodes that are powered by small irreplaceable batteries. These sensor nodes are densely deployed in the area to be monitored to sense and transmit data towards the base station. WSNs can greatly simplify system design and operation, as the environment being monitored does not require the communication or energy infrastructure associated with wired networks [1]. WSNs have great potential for many applications in scenarios such as detecting and tracking the passage of troops and tanks on a battlefield, monitoring environmental pollutants [2], habitat monitoring [3], classroom/home [4], structural monitoring [5], measuring traffic flows on roads, hazardous environment exploration and seismic sensing [6], tracking the location of personnel in a building and health monitoring.

The Wireless Body Area Network (WBAN) represents a recent evolution of sensor technology for the development of a new generation of Human-Computer Interfaces (HCIs) that provide natural and context-aware access to personalized services. Sensor technology enables the development of small and intelligent medical sensors which can be worn on or implanted in the human body. These biosensors are capable of measuring significant physiological parameters like heart rate, blood pressure, body and skin temperature, oxygen saturation, respiration rate, electrocardiogram, *etc.* The obtained measurements are communicated either via a wireless or a wired link to a central node, like a Personal Digital Assistant (PDA) or a microcontroller board, which may then in turn display the information on a user interface or transmit the aggregated vital signs to a medical center. Using a wired connection for this purpose turns out to be too cumbersome and involves a high cost for deployment and maintenance. However, the use of a wireless interface results in an emerging new technology called WBAN [7]. A WBAN could be seen as a special purpose wireless sensor network with a number of additional system design requirements. A WBAN is most likely to incorporate a wearable and implantable node operating in two different frequencies. An implantable node operates at 400 MHz using the MICS band whereas the wearable node could operate in ISM/UWB or some other band [8].

**Figure 1.**
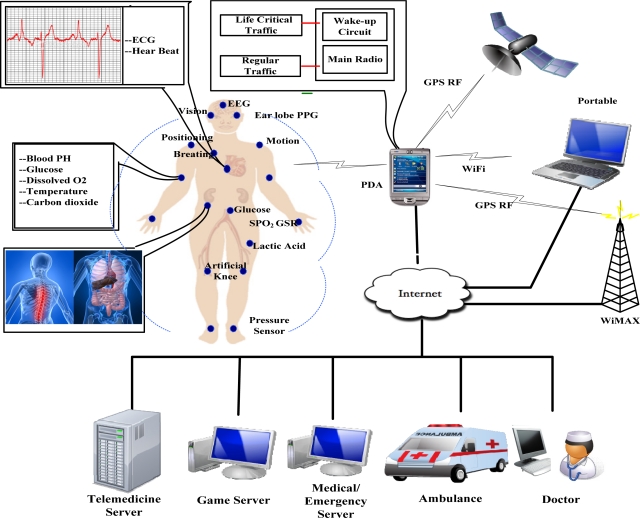
WBAN Architecture.

WBAN technology could provide the connectivity to support elderly people in managing their daily life and allows easy internetworking with other devices and networks, thus offering the health care worker easy access to a patient’s critical and non-critical data. A WBAN allows continuous monitoring of the physiological parameters. Whether the patient is in the hospital, at home or on the move, the patient will no longer need to stay in bed, but will be able to move around freely. Furthermore, the data obtained during a large time interval in the patient’s natural environment offers a clearer view to the doctors than data obtained during short stays at the hospital [9]. An example of a WBAN used for medical and non-medical applications to monitor the body function for sporting, health, entertainment, and emergency applications is shown in Figure 1 [8]. In the figure the game server is used for handling the non-medical applications like sports, while the other servers are used for dealing with medical related services. Several sensors are placed in clothes, directly on the body or under the skin of a person, which measure the temperature, blood pressure, heart rate, ECG (Electrocardiography), EEG (Electroencephalography), respiration rate, SpO2 levels *etc.* Limited energy resources are the primary constraint for WBAN to provide long term health monitoring.

Traditional MAC protocols are designed to maximize packet throughput, reduce latency and provide fairness but these lack energy conserving mechanisms, so when designing MAC protocols for a WBAN we must focus on reducing unnecessary energy expenditure due to wireless communication as well as insuring that the delay-sensitive data (emergency) are serviced within the application constraints.

Several energy conserving MAC protocols have been proposed based on contention or TDMA. The contention-based protocols also called the duty cycling protocols, such as BMAC [10], SMAC [11] and TMAC [12] *etc.* allow nodes to access a shared wireless medium independently. These protocols inherit good scalability and support topology changes but they are not suitable for WBAN due to the following reasons [13].

- The contention-based protocols use Clear Channel Assessment (CCA) to determine the status of the channel. However, the CCA for the implant device is not reliable. This is because path losses in tissue are much bigger than those in free space. All implant nodes with failed CCA become “hidden nodes” to the transmitter.
- The sleeping mechanism is complex, and it requires control overhead to keep neighbor nodes synchronized.
- Most of the WBAN traffic is correlated and a single physiological fluctuation triggers many sensors at the same time which will result in heavy collisions and extra energy consumption. For example, a patient suffering from fever triggers temperature, blood pressure, and respiration sensors at the same time.

In the TDMA-based protocols nodes are assigned their own time slot, and may access the shared medium only in this time slot. This allows for scheduling of sleep, idle listening and avoidance of collisions at the transceiver, without additional overhead. However, conventional TDMA schemes are not suitable for WBANs because these protocols require high quality time synchronization since the clock drift may lead to disastrous consequences.

Another approach is to use a radio triggered power management scheme which avoids useless wake-up periods. In this scheme a very low-power wake-up radio hardware component monitors the environment when the node enters sleep mode. When the stand-by radio transceiver receives radio signals, it wakes the sleeping node instantly, consequently saving energy spent in previous wake-up and listen intervals.

In this paper we propose a Very Low Power MAC (VLPM) protocol for WBANs. Our protocol is based on a radio triggered power management scheme. We categorize BAN traffic into two types: uplink (from BN to BNC) and downlink (BNC to BN). In the case of uplink traffic the BN sends a wake-up packet to the coordinator. The coordinator then assigns the resources to the node in the Res-ACK packet and receives the desired data. In the case of downlink traffic the coordinator sends a wake-up packet to the node along with the resources. The low power receiver of the BN then wakes up the processor to interpret the message and then wake-up the main radio to start the actual communication. To the best of our knowledge, this kind of low power approach has not been considered before for WBAN.

The rest of the paper is categorized into four sections. Section 2 presents related works. Section 3 briefly describes the proposed protocol. Section 4 presents useful analytical derivations and performance analysis of VLPM with other MAC protocols. The final section concludes our work.

## 2. Related Work

Owing to the fact that energy efficiency is a vital part of WBAN performance, recently there has been a considerable amount of research effort directed towards the development of low power energy efficient MAC protocols. The low data rate support and quick implementation of IEEE 802.15.4 has attracted many researchers. The performance of both beacon and non-beacon mode was studied in [14] and it was concluded that non-beacon mode is better than beacon mode in terms of throughput and latency. but comes with high power consumption costs. In [15] the authors supported the non-beacon mode for low data rate and asymmetric traffic. Li *et al.* considered the beacon enabled mode and achieve better performance in energy efficiency and latency by adjusting the super frame structure [16]. Although IEEE 802.15.4 is closely related to WBANs, it still does not provide enough solutions for communication among BAN nodes. A schedule-based MAC protocol called H-MAC is presented in [17]. This protocol uses heart rhythm information to perform synchronization and to improve energy efficiency, but the heart beat information is not always valid due to variations in the patient’s condition. A Dynamic TDMA protocol with focus on the dependability and power efficiency is presented in [18]. In Dynamic TDMA, the coordinator assigns slots to the nodes which have buffered packets and are released after transmission. However, this protocol still needs some form of synchronization which is difficult to achieve, especially in the case of implanted nodes. In BodyMAC [19], although the authors have defined downlink and uplink sub frames for on-demand and normal traffic but for implant node they have not considered a very low power wake-up mechanism. Marinkovic *et al.* presented an energy efficient MAC protocol to support streaming of large amounts of data in WBANs with little amount overhead and no idle listening [20]. In TaMAC [21], the authors tried to solve the idle listening and overhearing problems by taking into account the traffic information of the sensor nodes to dynamically adjust the duty cycle of the sensor nodes according to their traffic-patterns.

Some of the most important design constraints of a node in medical applications are the response time along with energy efficiency. For pacemakers and in control systems, long latencies can lead to a huge loss of human life and system instability. Techniques are therefore needed to save energy as well as to reduce latency. One of the possible alternatives to reduce latency and to improve energy efficiency is to use an additional low power wake-up radio hardware which is capable of reacting instantly on an event of interest and with negligible power consumption. The use of wake-up radio hardware in WSNs is not new. PicoRadio [22], one of the earliest works on wake-up radio, uses a carefully designed very low power transceiver module, which is capable of monitoring the radio environment. In [23] the authors have shown that use of wake-up radio is very beneficial in terms of energy consumption rather than in low duty cycling protocol. Gu *et al.* proposed a radio triggered wake-up circuit to control the operation of sensor node and to eliminate the energy wastage of wake-up radio without addressing capabilities, thus restricting the functionality significantly [24]. Doorn *et al.* [25], proposed a prototype to work in the 868MHz band, for achieving energy efficiency with the help of a low cost radio triggered wake-up. Due to the lack of addressing mechanism in their proposed prototype, energy is wasted by waking up all the other nodes unnecessarily and hence it is not suitable for WBANs. In [26], the authors have proposed to attach passive RFID (Radio-frequency Identification) tags to the sensor node for conserving node energy. But to activate a passive RFID tag we need to have an RFID reader to send strong radio signals. In [27] the authors proposed a radio triggered wake-up with addressing capabilities that allows the non-target nodes to go to sleep mode quickly just after interpreting the ID in the wake-up packet.

## 3. Protocol Description

In this section, we present the architecture of low power wake-up receiver/transmitter, the network model and principal of operation of VLPM.

### 3.1. Hard Ware Architecture of the Wake-Up Receiver/Transmitter

There are several types of radio triggered circuits, but for a WBAN we would prefer to use a stored energy radio triggered circuit with a filter. The reason for this circuit to use is that as in the WBAN the transmitting power and the receiving antenna gain is normally very limited and the chances of tissue loss and other path losses are greater. Thus the energy received from a signal by the simple radio triggered circuit may not be enough to trigger the interrupt in all the cases.

**Figure 2.**
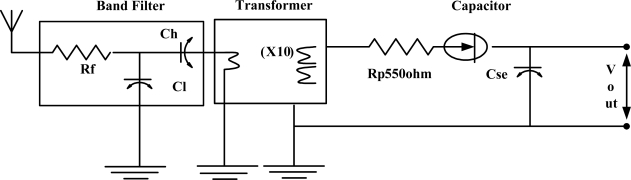
Wake-up receiver schematic.

Figure 2 shows the schematic of the radio triggered wake-up receiver with filter. This stand-by hardware first collects and stores the energy from the radio signals and then generates a sufficient output voltage to interrupt the MCU [24].

The job of the antenna of the radio triggered circuit is to collect energy from electromagnetic waves. The incoming radio frequency activates the antenna and then the electrical energy of the antenna powers other parts of the circuit and produces an output voltage. The use of a band filter which is made up of a resistor (Rf) and capacitors (Ch, Cl) intensifies the selecting power of the circuit to distinguish between wake-up radio signal frequency and false alarms. The transformer X10 is used to increase the input voltage on the antenna. The transformer makes it easier to reach the voltage threshold required for triggering an interrupt. The loss during the voltage transforming process is negligible. As compared to the basic radio triggered circuit the store energy radio triggered circuit requires some time *i.e.*, about 2.8 ms is needed to accumulate energy for generating enough voltage to trigger the interrupt [24]. This delay increases with an increase in distance. Since in WBAN as nodes normally work within a 3 meter range, therefore we believe that this delay will not increase more.

**Figure 3.**
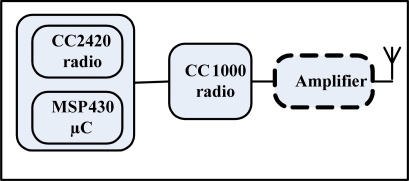
Wake-up transmitter block diagram.

The capacitor Cse is used to store energy. As soon as the antenna receives radio signals, the energy is accumulated on Cse due to the flow of current which causes the voltage to increase across the capacitor. The wake-up signal transmitter used in our work consists of a TelosB node (using CC2420 as main radio and MSP 430 as MCU), Texas Instruments Inc.’s CC1000 radio transceiver and an optional ZHL-2010 frequency amplifier for increasing communication range as shown in Figure 3 [27]. The CC1000 radio generates a modulated signal which has the capability to uniquely address different nodes and also to send short commands within the wake-up packet.

### 3.2. Network Model

This sub-section describes the proposed network model. The network is composed of a BNC (BAN Coordinator) and BNs (BAN Nodes). The BNC can either be a standalone device or integrated within another device such as a cell phone or a Personal Data Assistant (PDA) whose job is to obtain the data from BNs and send it onward for further processing. The BNs are battery powered sensor nodes which measure the physiological parameters and send to the BNC. Both the BNs and BNC consists of a low power wake-up transmitter as well as a receiver.

**Figure 4.**
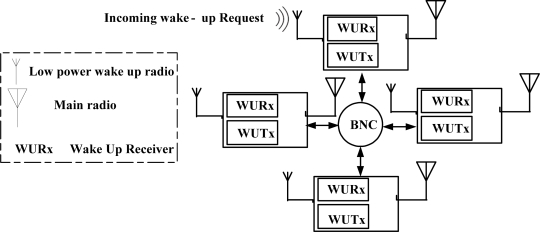
Network model.

The wake-up receiver monitors the channel for activity constantly and keeps the MCU along with main radio in sleep mode. When an event of interest happens, the wake-up receiver then interrupts the MCU, which then wakes up the main radio to start communication. Figure 4 shows the proposed network model.

### 3.3. Principle of Operation

Most of the conventional power management schemes are based on periodic wake-up/sleep schedules. Nodes wake-up periodically to listen for incoming packets and then decide whether to go into a sleep state or stay awake. In the case, if no packets are destined to them the nodes then set the timer for the next wake-up and switches off its radio. Idle listening dominates the total energy consumption in this case. Furthermore, in the wake-up/sleep protocols the microprocessor always remains in wake-up mode which also has a negative effect on the network life time. Another problem is that if we decrease the duty cycle we will save energy but we will have to pay the penalty for latency. For time-sensitive applications like pacemakers and control systems long latencies can lead to system instability. To keep the network responsive, the wake-up period needs to happen frequently so that the nodes wake-up quickly and reply to the incoming messages. Due to the unpredictability and low traffic, a majority of wake-up periods may initiate in vain, which results in a waste of energy.

**Figure 5.**
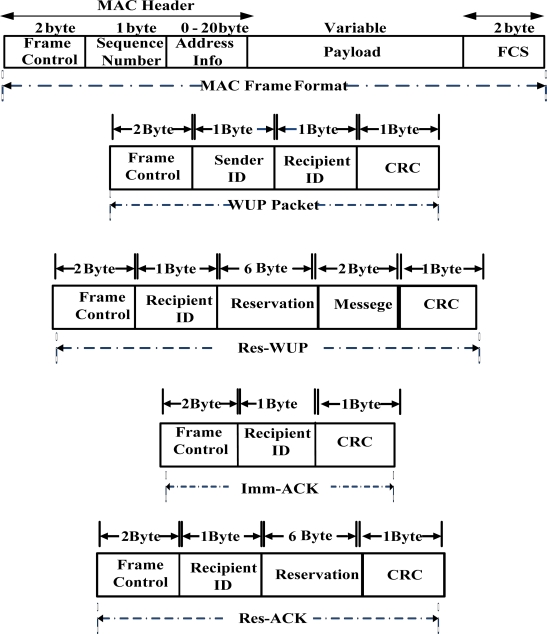
The packet formats of the proposed protocol.

In the proposed scheme a radio triggered wake-up receiver circuit is attached to a sensor node which keeps the main radio & MCU in sleep mode until it receives a WUP (Wake-up Packet) on the wake-up radio. After receiving a WUP, the MCU is activated for interpreting the command. After interpretation the MCU then decides whether to go to sleep or activate the main radio based on the address field in the wake-up packet. Unlike duty cycling protocols, our protocol works on an “on-demand” bases *i.e.*, nodes are only awoken when they are the intended senders or receivers of data packets. Our protocol assumes two different types of wake-up and ACK packets namely WUP, Res-WUP and Imm-ACK, Res-ACK respectively. The BNC uses Res-WUP and Res-ACK by piggybacking resource allocation on wake-up and acknowledgement messages. This piggybacking becomes rather difficult and more complex when used for applications in which a message is needed to be sent out every few seconds, or even minutes. But in the case of WBAN where the traffic is low and infrequent, this piggybacking approach is more beneficial in terms of energy utilization and latency. In VLPM, Imm-ACK is used by the BN as well as by the BNC for simple acknowledgment without resource allocation. Similarly WUP is used by the BN to inform the BNC about the emergency event. The wake-up packet is sent at a special radio frequency which does not interrupt with other types of radio communication. Figure 5 shows the proposed frame formats. Upon receiving the WUP, the circuit interrupts the MCU to interpret the message. The MCUs of the non-targeted nodes process the incoming message and then turn off quickly while the MCU of the addressed node turns on its main radio for radio communication.

### 3.4. Handling Downlink and Uplink Traffic

In the case of downlink traffic the BNC requests the data from the BNs. Normally the BN does not know when BNC will need the data, so in the earlier approaches the BN has to use a wake-up/sleep approach on a periodic basis. In the wake-up period, the BN wakes up for a certain amount of time to sense the channel for activity and then either goes to the sleep mode again or remains awake. This periodic wake-up and sleep approach leads to idle listening and over hearing problems.

During idle listening, the transceiver of the BN remains in an active state which consumes a lot of energy. When the BN goes into sleep mode then it remains in that mode until its scheduled wake-up. In the meantime, if a sender wants to send a packet to that BN then the sender will have either to wait till the wake-up time of that node or will have to send a lengthy preamble followed by the desired data packet. Both cases results in a huge amount of energy consumption as well as in undesirable latency. In some applications of WSN this latency may not be so dangerous but in some applications such as occurrence of emergency events, this latency can lead to critical and potentially life-threatening situations.

**Figure 6.**
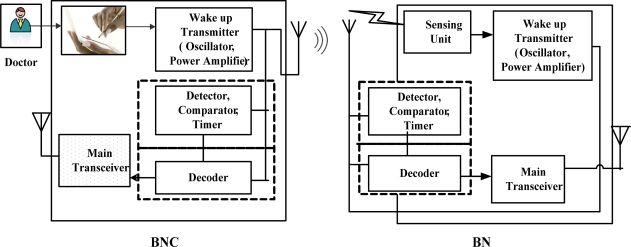
Low power downlink communication.

Our protocol can respond well to the above mentioned problems of traditional MAC protocols. In our proposed protocol, in the case of downlink traffic the coordinator (BNC) reads the command from the quarter it is concerned with and then acts according to the following procedure. The BNC sends an out-of band modulated signal (Res-WUP) to the BN. The Res-WUP contains the desired node ID along with other necessary information like resource allocation, type of data needed *etc.* Upon receiving the Res-WUP, the wake-up receivers of all non-targeted BNs in the range go to sleep mode just after interpreting the message. In the interpretation process, only MCU is in active mode while the main transceiver remains in sleep mode. Therefore simple message interpretation is required which is not overly expensive in terms of energy usage. The MCU of the destined node wakes up other hardware components including the main radio. The node then sends the data packet and gets Imm-Ack. Figure 6 describes the downlink communication between BNC and BN.

**Figure 7.**
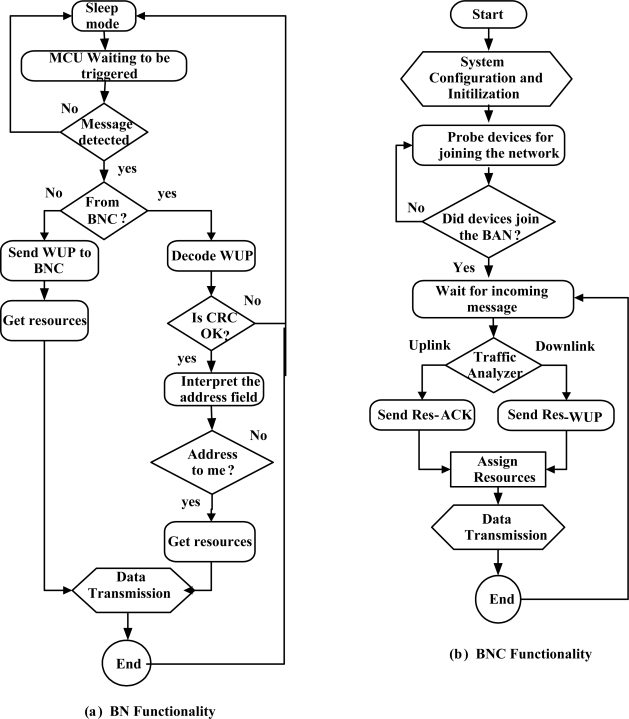
Flowchart describing VLPM Functionality.

Similarly in the case of uplink traffic emergency signal in the form of a WUP arrives from the BN to the BNC, when a life critical sensor node senses some abnormality in its relevant part of body. When the emergency event occurs, the sensor quickly triggers the wake-up transmitter, which prepares a wake-up packet by including a node ID and sends it to the coordinator. The wake-up receiver of the coordinator will interrupt the MCU. The MCU will then wake up its main radio if not already awake and will send Res-ACK to the destination node (BN). The BN then sends the data and gets Imm-ACK from BNC. The BNC then process the data and forwards it onward for further necessary action to doctors, hospitals and databases. The advantage of our proposed protocol is that the wake-up signal will not interrupt the ongoing communication between the coordinator and other nodes during the transmission of the wake-up signal. Also, due to the mode always being on a low power wake-up receiver circuit, the destination is informed in time with very low power without using long preamble or without using complex synchronization. The flow charts in Figure 7 shows the basic functionality of the BNC and BN *i.e.*, how the wake-up receiver, transmitter and other circuitry works.

## 4. Performance Evaluation

This sub-section describes the evaluation of radio triggered circuit for WBAN and the analytical models used for comparing VLPM with other MAC protocols.

### 4.1. Evaluation of the Radio Triggered Circuit for WBAN

In this section we evaluate the effectiveness of the radio triggered circuit for WBAN in terms of antenna gain, latency and lifespan for different BAN traffic scenarios. We are taking the same scenario as that described in [24] instead of taking a specific BAN chip because in our approach the radio triggered circuit makes no use of the node battery power. Therefore its performance will be similar to any chosen BAN RF chip. According to our scheme, each BN will either be in active mode or in sleep mode. In sleep mode all the hardware components except the interrupt controller and low power amplifier switch off.

**Figure 8.**
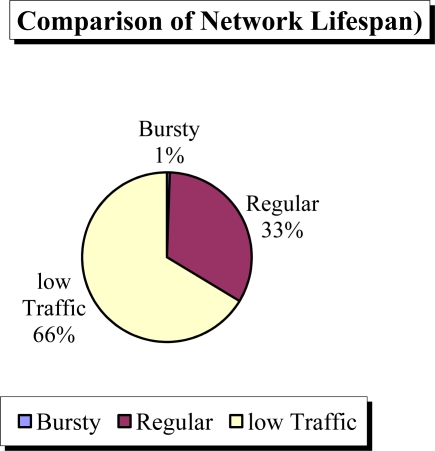
Comparison of network Lifespan.

In this scenario, we assume that there exists one event per two BAN hours and the event lasts for 60 BAN seconds. We also assume that each BN uses two 2000 mAh AA batteries with an average voltage of 1.5 V, during the network life time. The current drawn is 0.01 mA and 20 mA in sleep and active mode respectively. The BN takes 5 ms to go from sleep mode to active mode using 30 mA current. We computed the lifespan of the network for regular, low traffic (on-demand) and sporadic bursts of traffic as shown in Figure 8.

In the case of regular traffic we assume that the BN wakes up for 200 ms (including switching time) to communicate with BNC and then goes to sleep mode for 1 BAN minute (the BN wakes up for event detection after every 60 seconds and then remains in wake-up mode for 200 ms in total to transfer the detected data to the BNC). This schedule repeats continuously. In the case of on-demand traffic, the BN remains in the sleep mode and wakes up only when the BNC requires it to send the data by sending a low power wake-up call. For bursty traffic all the nodes need to be awake all the time throughout the network life time.

The network lifespan for regular traffic (L_reg_) can be computed as:

(1) $$\begin{array}{l} 2,000\;\text{mAh }*2*1.5V=\\ \{L_{\text{reg}}*(((60,000\;\text{ms-}5\;\text{ms})*0.1\text{ mA }+5\text{ ms }*30\text{ mA }+200\text{ ms }*20\text{ mA})*2\;*1.5\text{ V})*\\ \left(85680/60.2\right)\\ +L_{\text{reg}}*((60,000\;\text{ms }*20\text{ mA }+5\text{ ms }*30\text{ mA})*2*1.5V)*\;12\} \end{array}$$

solving this Equation we get L_reg_ = 249.52 days.

similarly the network lifespan for on-demand traffic (L_on-dmnd_) can be computed as:

(2) $$\begin{array}{l} 2,000\text{ mAh }*2*1.5V=\\ \left\{L_{\text{on-dmnd}}*(12\;*\;((60,000\;\text{ms-}5\;\text{ms})*\;20\text{mA }*2*\;1.5\;\text{V }+5\text{ ms }*\;30\text{ mA }*\text{ 2 }*1.5\text{ V})\right)\\ +L_{\text{on-dmnd}}*((1-0.833\%)\;*0.1\text{ mA }*2*\;1.5\text{ V})\} \end{array}$$

Solving Equation (2) for L_on-dmnd_ we obtained lifespan of 449.98 days.

For bursty traffic the network lifespan is:

(3) 2,000 mAh =20 mA =4.17 days

In order to trigger the interrupt for MCU, we need enough energy in the receiving signal which is in the form of electromagnetic waves. This signal energy depends on the transmitting power of the sender and/or the receiving and transmitting antenna gains. The signal strength decreases when the distance increases. To handle this problem either we need to increase the transmitting power or the receiving antenna gain. In the case of WBAN we cannot increase the transmitting power above a certain limit, because of concerns over power consumption and radio interference. The received power depends on Effective Isotropic Radiated Power (EIRP), receiving antenna gain, path losses and the distance between the sender and receiver.

The received power (P_rx_) at the antenna can be calculated as:

(4) $$P_{\text{rx}}=(P_{\text{tr}}*G_{t}*G_{r})/(L_{p})\;\;\text{Where }L_{p}={\left(4*R*\pi \right)}^{2}/{\left(\lambda \right)}^{2}$$

Here P_tr_ is the transmission power, G_t_ is the transmitting antenna gain, G_r_ is the receiving antenna gain, L_p_ is free space path loss, R is the distance between transmitter and receiver and λ is the wavelength.

We used the example of a Mica2 mote with a CC1000 radio transceiver. This radio can transmit radio signals from −10 dBm to +10 dBm [24]. We assume P_tr_ = +10 dBm, R = 3 m, G_t_ = 6.31 mW, G_r_ = 3.98 mW and λ = 0.69 m. By putting these values in the formula we get P_rx_ = 0.084 mW which is enough to generate the desired voltage (0.6 V [24]) for triggering the interrupt.

**Figure 9.**
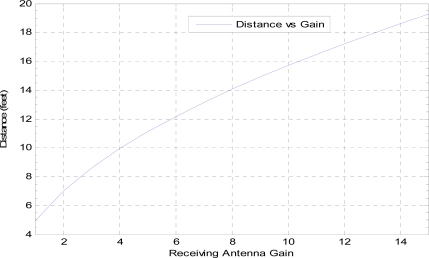
Receiving antenna gain *vs.* distance.

Figure 9 shows that as the distance between the nodes increases, the need for the antenna of higher gain also increases. However using an antenna of higher gain is not practically applicable in the case of WBAN especially for implanted nodes. Therefore, in the case of WBAN, where the transmitting power as well as antenna gains is limited, we need to store some energy to trigger the interrupt successfully.

### 4.2. Analytical Models for Power Consumption and Latency

In this section, we provide models for latency and power consumption of VLPM and compare them to the corresponding models of B-MAC, X-MAC, TMAC and SCP-MAC. To obtain numerical results we are considering TeloseB platform with T1 CC1000 transceiver [28]. Table 1 shows the parameters used and their values [28]. The performance models are based on analysis presented in [29].

For deriving the performance models we assume that: (1) the channel is an ideal channel without errors; (2) There is no packet loss due to the buffer overflow at the receiving node; (3) at any packet arrival interval, there is one sensor sample and the data is sent directly to the one hop neighbor (BN or BNC); (4) there is no contention; (5) the power consumption of idle listening is equal to that of power receiving; (6) the time and power needed to switch from Tx to Rx and *vice versa* are same.

Data exchanges are normalized to packet generation time (T), during which all nodes generate exactly one data frame. Average power consumption for each protocol can be calculated by multiplying the power consumed during the activity (transmission, reception and sleep) and its duty cycle (duration during which the concerned activity performed) as shown in Equation (5). We have computed the D_tx_ and D_rx_ for each protocol in its corresponding sections.

Latency is the second performance metric of interest. It is bounded by application requirements, for example, in the case of an emergency; event detection must be reported at the BNC within a few seconds. Depending on the MAC protocol there can be a rather large difference between average latency and worst-case latency. We therefore only model the average latency of the MAC protocols concerned:

(5) $$P_{\text{avg}}=P_{\text{tx}}*D_{\text{tx}}+P_{\text{rx}}*D_{\text{rx}}+P_{\text{sleep}}*D_{\text{sleep}}$$

where D_sleep_ = (1 – D_tx_ – D_rx_)

#### X-MAC

In the X-MAC the receiver wakes up periodically to listen for the preamble and the transmitter continuously transmits strobed preambles (send small bursts then waits for ACK then sends another preamble and so on). The receiver needs to listen to at least two preambles and one ACK before starting the data communication with the transmitter.

The values of D_tx_, D_rx_ and L_avg_ can be calculated as:

(6) $$D_{\text{tx}}=(\text{NP }*\;T_{p}+T_{\text{set}}+T_{D})/T$$

(7) $$D_{\text{rx}}=(T_{\text{lpl}}/T_{w})+((\text{NP}+1)T_{\text{ack}})/T$$

where NP and T_D_ means average number of preambles transmitted and data transmission time of a data packet respectively:

(8) $$\text{NP }=\;T_{W}/2(T_{p}+T_{\text{ack}})\;\;\text{and }T_{D}=L_{\text{pay}}/R$$

(9) $$L_{\text{avg}}={2T}_{p}+{2T}_{\text{ack}}+T_{w}/2+T_{D}+{2T}_{\text{set}}$$

#### B-MAC

B-MAC utilizes low power listening and an extended preamble to achieve low power communication. Nodes sleep, wake up and perform channel sensing periodically. Each node can have an independent schedule. The transmitting node precedes the data packet with a preamble that is greater or equal to the longest wake-up period of any node. A node samples the medium during the listening period and if a preamble is detected it remains awake to receive the data. The purpose of using the lengthy preamble is to assure the sender that at some point during the preamble the receiver will detect the preamble, and remain awake to receive the data. The values of Dtx, Drx and Lavg can be calculated as:

(10) Dtx =(Tset + Tw + TD)/T

(11) Drx=((Tset+Tcca)/Tw)+{((Tw/2+TD−Tcca)*N)+(Tset+Tack)}/T

(12) Lavg=Tw+Tack+TD+Tset+Tcca

#### T-MAC

T-MAC is the improved version of S-MAC by shortening the awake period if the channel is idle. In T-MAC the nodes listen to the channel for only a short time (TA) after the synchronization phase and then return to sleep mode if no data is received. In the case of data, the node remains awake till data reception or untill the end of the awake period. The values of D_tx_, D_rx_ and L_avg_ for T-MAC can be calculated as:

(13) $$D_{\text{tx}}=((T_{D}+T_{\text{RTS}}+2T_{\text{set}})/T)+(T_{\text{set}}+T_{B})/T_{\text{sync}}$$

(14) $$\begin{array}{c} D_{\text{rx}}=((2T_{\text{set}}+T_{\text{cw}}+T_{\text{RTS}})/T_{W})+(((2T_{\text{set}}+T_{\text{cw}}/2+T_{\text{CTS}})*N)/T)\\ +((2T_{\text{set}}+T_{\text{CTS}}+T_{\text{ack}})/T)+((T_{\text{set}}+T_{\text{cw}}-T_{B}/2)/T_{\text{sync}}) \end{array}$$

Average latency can be calculated as:

(15) $$L_{\text{avg}}=T_{w}+T_{\text{ack}}+T_{D}+T_{\text{set}}+T_{\text{cca}}$$

#### SCP-MAC

This MAC protocol uses a short wake-up tone instead of a long preamble. Sync packets are piggybacked with data frames. It uses two contention windows with T_cw_/4 average back off time. The receiver normally receives half of the wake-up tone and the second contention window. The T_tone_, D_tx_, D_rx_ and L_avg_ can be calculated as:

(16) $$T_{\text{tone}}=((4⊖T)/(N))+T_{\text{cca}}$$

(17) $$D_{\text{tx}}=(T_{\text{sp}}+T_{D}+T_{\text{tone}}+{2T}_{\text{set}})/T$$

(18) $$\begin{array}{c} D_{\text{rx}}=((T_{\text{set}}+T_{\text{cca}})/T_{w})+((T_{\text{tone}}/2+T_{\text{cw}}/4+T_{\text{cca}}+{3T}_{\text{set}}+T_{\text{sp}}+T_{D})*N)/T\\ +({2T}_{\text{cca}}+T_{\text{ack}}+{3T}_{\text{set}})/T \end{array}$$

(19) $$\text{Lavg}=T_{w}/2+T_{\text{ack}}+T_{D}+4⊖T_{\text{sync}}+T_{\text{cca}}$$

#### VLPM

**Figure 10.**
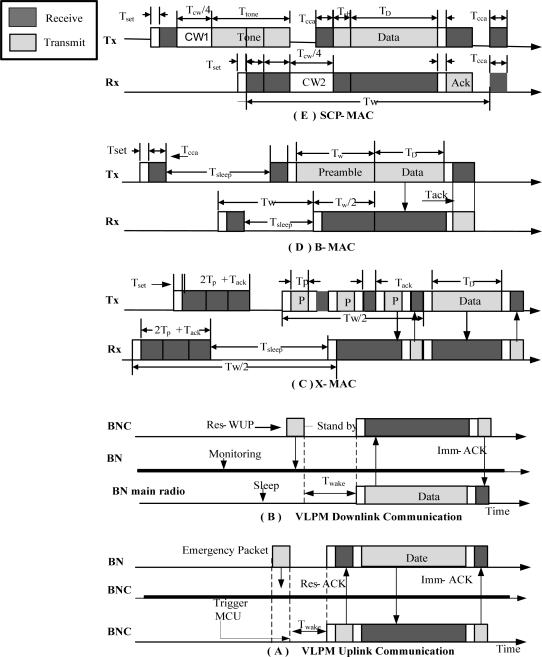
Principle of operation of different MAC Protocols.

In VLPM unlike the above mentioned conventional MAC protocol, the main radio never takes part in low power listening activities. Channel monitoring is performed by a low power wake-up receiver. Figure 10 shows the radio activities of VLPM for both uplink and downlink traffic along with other protocols. The average time required by the low power transmitter to transmit a single bit is 530 μs and the total power consumption of a node in the sleep mode *i.e.*, P_sleep_ = 12.528 μW [27]. We assume that the address decoding time is negligible. The D_tx_, D_rx_ and L_avg_ for uplink traffic can be calculated is:

(20) $$D_{\text{tx}}=(T_{\text{wup}}+{2T}_{\text{set}}+T_{D})/T$$

(21) $$D_{\text{rx}}=(T_{\text{wake}}+T_{\text{Res-ack}}+T_{\text{set}}+T_{\text{imm-ack}})/T$$

(22) $$\begin{array}{c} P_{\text{avg}}=((P_{\text{txbn}}*T_{\text{wup}})+({2T}_{\text{set}}*P_{\text{set}})+(T_{D}*P_{\text{tx}}))/T+\\ ((P_{\text{wake}}*T_{\text{wake}})+(T_{\text{Res-ack}}+T_{\text{set}}+T_{\text{imm-ack}})*P_{\text{rx}})/T+\\ ((1-D_{\text{tx}}-D_{\text{rx}})*P_{\text{sleep}})/T \end{array}$$

(23) $$L_{\text{avg}}=T_{\text{wup}}+{3T}_{\text{set}}+T_{D}+T_{\text{wake}}+T_{\text{Res-ACK}}+T_{\text{imm-ack}}$$

Similarly for down link traffic we can calculate D_tx_, D_rx_ and L_avg_ as:

(24) $$D_{\text{tx}}=(T_{D}+T_{\text{set}}+T_{\text{wake}})/T$$

(25) $$D_{\text{rx}}=(T_{\text{Res-wup}}+{2T}_{\text{set}}+T_{\text{imm-ack}})/T$$

(26) $$\begin{array}{c} P_{\text{avg}}=((P_{\text{txbn}}*T_{\text{wup}})+(T_{\text{set}}*P_{\text{set}})+(T_{D}*P_{\text{tx}}))/T+\\ ((P_{\text{wake}}*T_{\text{wake}})+(T_{\text{Res-ack}}+{2T}_{\text{set}}+T_{\text{imm-ack}})*P_{\text{rx}})/T+\\ ((1-D_{\text{tx}}-D_{\text{rx}})*P_{\text{sleep}})/T \end{array}$$

(27) $$L_{\text{avg}}=T_{\text{wake}}+{3T}_{\text{set}}+T_{\text{Res-wup}}+T_{D}+T_{\text{imm-ack}}$$

### 4.3. Results and Discussion

**Figure 11.**
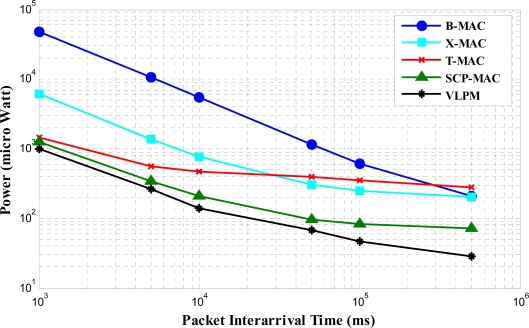
Comparison of energy consumption.

Using the parameter values specified in Table 1, we found the average power consumption for low rate platform utilizing CC1000 transceiver [28]. The results in Figure 11 show that VLPM outperforms both synchronized and unsynchronized protocols in terms of energy consumption. Due to the long preamble in B-MAC, its energy consumption is the highest of all. In B-MAC a sender needs to transmit the preamble for the duration of the longest wake-up period of any node, while the receiver and its neighbors listen on an average of half of the preamble.

The reduced energy consumption in X-MAC than B-MAC is due to the fact that the average length of the strobed preamble is half of the wake-up period (Tw). The replacement of long preamble with a short wake-up tone in SCP-MAC which wakes the sender and receiver almost at the same time results in much better energy saving.

For the packet generation time from 1 second to 1,000 seconds, the power consumption of the BNs in VLPM is from 40.68 μW to 853.44 μW. The reasons for this lowest power consumption is that, in VLPM the main radio turns on just before the actual data communication. There is no need to send a lengthy preamble and the non-targeted nodes go back to sleep soon just after processing the address info in the wake-up packet, without activating the main radio.

**Figure 12.**
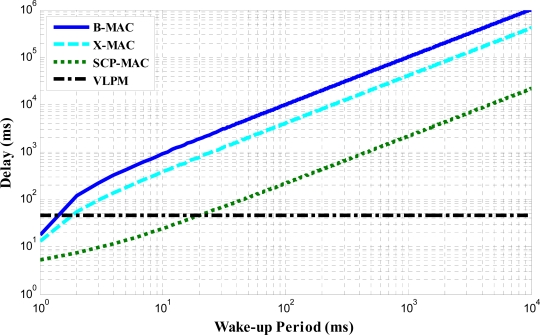
Delay comparison of VLPM with asynchronous MAC protocols.

Figure 12 shows the latency comparison of VLPM with other protocols. The latency in the case of uplink traffic depends on WUP, Res-ACK and the data packet. There is no dependency on the wake-up period because the low power receiver is always in a channel monitoring mode. Unlike VLPM, the latency in the other protocols strongly depends on the wake-up period. Our observation is justified by the observations made by Kerl *et al.* that, latency is affected by wake-up period while power consumption is affected by duty cycle [30]. VLPM incurs latency orders of magnitude lower than that of X-MAC, B-MAC and SCP-MAC. We further observe that for a wake-up period less than 20 ms, X-MAC and B-MAC dominates, but such small wakes up periods are not typical. Also lower wake-up periods mean higher duty cycles which lead to higher energy consumption.

## 5. Conclusions

We have proposed a very low power MAC protocol for WBANs. This protocol helps the BAN reduce idle listening and improve energy efficiency as well as response time. We have carried out comparative studies with X-MAC, B-MAC, T-MAC and SCP-MAC. Our protocol dominates in terms of both energy efficiency and response time. The high energy usage and latency in X-MAC, B-MAC, T-MAC and SCP-MAC is due to the fact that these protocols require the node to wake up periodically to monitor the channel and then go to sleep mode if there is no activity for that node to do. On the other hand our protocol reduces energy consumption by keeping the node along with the main radio in sleep mode all the time when there is no need for communication and improves response time by instantly waking up the desired node when it is needed. The use of a low power wake-up receiver minimizes the overhead incurred by time synchronization and by sending long preambles. An identity based wake-up receiver reduces overhearing up to a great extent, because only the intended nodes wake up and take part in communication while all the other nodes go quickly to sleep mode after interpreting the wake-up packet. The energy consumption of the non-target nodes in interpreting the message is negligible.

## Figures and Tables

**Table 1.** Model Parameters.

**Parameter**	**Description**	**Value**	**Parameter**	**Description**	**Value**
⊖	Clock drift	20 ppm	P_tx_	Transmit power of main radio	29.9 mW
R	Bit rate	76.8 Kbps	P_sleep_	Sleep power	37 μW
T_ack_,T_CTS_, T_RTS_, T_P_	Duration of control packets	0.83 ms	T_sync_	Sync time	90 s
T_B_	Beacon Length	3.33 ms	T_cca_	CCA time	256 μs
L_pay_	Payload length	32 bytes	T_wake_	Wake-up time	1.5 ms
N	Number of nodes	10	T_s`et_	Time required for switching from Tx to Rx and vice versa	130 μs
P_rx_	Receive power of main radio	24.5 mW	P_wake_	Power required to wake up main radio	855 μW
P_set_	Power to go to Tx or Rx mode	27.2 mW	T_lpl_(X-MAC)	Time for low power listening	2.88 ms
T	Packet generation time	Variable	T_sp_(SCP-MAC)	Time for sync packet	0.03 ms
T_wup_	Duration of WUP	21.2 ms	T_Res-ack_	Time for Piggybacked ack	1.04 ms
P_txbn_	Transmit Power of Wake-up radio	1.6 mW	P_rxbn_	Receive Power of wake-up radio	0.4 mW
T_Res-wup_	Duration of Piggybacked WUP	1.25 ms	T_imm-ack_	Duration of immediate ack	16.9 ms
